# Nanosilver Environmental Safety in Marine Organisms: Ecotoxicological Assessment of a Commercial Nano-Enabled Product vs an Eco-Design Formulation

**DOI:** 10.3390/toxics13050338

**Published:** 2025-04-25

**Authors:** Arianna Bellingeri, Analía Ale, Tatiana Rusconi, Mattia Scattoni, Sofia Lemaire, Giuseppe Protano, Iole Venditti, Ilaria Corsi

**Affiliations:** 1 Department of Physical Sciences, Earth and Environment, University of Siena, 53100 Siena, Italy; arianna.bellingeri2@unisi.it (A.B.); tatiana.rusconi@outlook.com (T.R.); scattoni4@student.unisi.it (M.S.); giuseppe.protano@unisi.it (G.P.); ilaria.corsi@unisi.it (I.C.); 2 Department of Toxicology, Pharmacology and Legal Biochemistry, Facultad de Bioquímica y Ciencias Bioógicas (FBCB-UNL), CONICET, Santa Fe 3000, Argentina; 3 Department of Sciences, Roma Tre University of Rome, 00146 Rome, Italy; sofia.lemaire@uniroma3.it (S.L.); iole.venditti@uniroma3.it (I.V.)

**Keywords:** silver nanoparticles, capping, ecotoxicology, nano-enabled products, eco-design

## Abstract

With the increasing use of manufactured nanomaterials in consumer products, especially silver nanoparticles (AgNPs), concerns about their environmental impact are rising. Two AgNP formulations were tested, the commercial nanosilver product nanArgen™ and a newly eco-designed bifunctionalized nanosilver (AgNPcitLcys), using marine organisms across three trophic levels, microalgae, microcrustaceans, and bivalves. Acute toxicity was assessed on the diatom *Phaeodactylum tricornutum*, brine shrimp larvae *Artemia franciscana*, and bivalve *Mytilus galloprovincialis*. The behavior of the formulations in marine media, including stability across a concentration range (0.001–100 mg/L), was also evaluated. Results showed that nanArgen™ was less stable compared to AgNpcitLcys, releasing more silver ions and exhibiting higher toxicity to microalgae (100% growth inhibition at 1 mg/L) and microcrustaceans (>80% mortality at 10 mg/L). Conversely, AgNPcitLcys (10 µg/L) was more toxic to bivalves, possibly due to the smaller nanoparticle size affecting lysosomal membrane stability. This study highlights how eco-design, such as surface coating, influences AgNP behavior and toxicity. These findings emphasize the importance of eco-design in minimizing environmental impacts and guiding the development of safer, more sustainable nanomaterials.

## 1. Introduction

The continuous use of manufactured nanomaterials (MNMs) in several commercial sectors, particularly consumer products (e.g., drugs, food, textiles, and cosmetics), causes a significant release in wastewater, with serious implications for freshwater and marine water contamination [1,2,3,4]. While nanosilver’s environmental concentrations are predicted to be in the low range of <1 µg/L [5], it is among the most used MNMs, and, as a consequence of the COVID-19 pandemic, it has reached an accelerating trend of production, which is expected to increase up to 2030 [6,7,8]. Additional nanosilver applications, such as in the development of new sensors or as biocide for wastewater treatment, are increasing despite the hazard posed to aquatic living beings [9,10,11,12,13,14,15].

The ecotoxicity of nanosilver has been widely documented for aquatic species, including bioaccumulation and trophic transfer along food webs [16,17,18,19,20]. Nanoscale dimensions, as well as silver ion dissolution, have been indicated as responsible for the observed hazard [18,21,22].

The surface functionalization of silver nanoparticles (AgNPs) drives their behavior and environmental stability, including dissolution, with the release of silver ions in aqueous solution and consequent ecotoxicity [23,24,25,26,27]. Several coating molecules have been identified as protective towards nanosilver ecotoxicity and responsible for controlling physicochemical properties, such as, for instance, agglomeration/aggregation and/or enhancing their biocompatibility and bioavailability [28,29]. The role of particle coatings in driving nanosilver behavior and ecotoxicity has recently been shown by our findings, in which freshwater and marine model species were exposed during acute and long-term scenarios, resulting in various degrees of ecological risk [30,31], and by numerous other studies [32,33,34].

Although the dynamics of commercial products compared to bare MNMs are still unclear, little attention has been paid to nanosilver byproducts and their ultimate ecotoxicity for aquatic life. The stability of AgNPs in aqueous suspensions represents a critical element for the sustainable and safe design of nanosilver [22,35]. Designing MNMs that maintain their specific properties while respecting environmental safety and environmental sustainability (e.g., criteria established through life cycle assessment) is defined as a priority and will guide the nanotechnologies of the future, at least in the European context [36,37,38]. An eco-design based on the safety of nanosilver from an ecotoxicological point of view is in line with this strategy and requires further development and implementation [39].

Our previous investigations showed that a nanosilver-enabled consumer product results in similar accumulation and toxicity of commonly tested AgNPs in the marine mussel *Mytilus galloprovincialis* [40]. However, there are scarce and fragmentary scientific literature reports on nanosilver-enabled products’ ecotoxicity, thus limiting a proper ecological risk assessment for aquatic wildlife and the work of regulatory agencies [41].

In the field of nanotoxicology, there is a need for both novel synthesis of NMs with eco-designs, which bridge the gap in safety [42], and the “green toxicology” concept in terms of animal welfare [43]. In this sense, experiments with microorganisms have been recommended for their critical role in trophic webs and high reproduction rate, while filtering animals like mussels have gained importance for being particularly threatened by nanopollution given their non-selecting filter and suspension feeding habits [44]. As these marine organisms have been poorly assessed in comparison with freshwater ones (not only for AgNPs but also for most of the MNMs) [5,42], the novelty of this work lies in testing a variety of group of marine invertebrates with different sensitivity to the potential hazard of this emerging pollutant through exposure to conventional (commercially available) and eco-designed AgNP formulations. Furthermore, the complete characterization of both AgNPs provides an environmentally relevant perspective in terms of nanoparticle behavior and fate.

Moving on from our previous results, the present study investigated the ecosafety of the commercial formulation of nanosilver named nanArgen™ and a new eco-designed batch of AgNPs bifunctionalized with citrate and L-cysteine (AgNPcitLcys) for marine ecotoxicity. Acute ecotoxicity assays were conducted using the marine microalgae *Phaeodactylum tricornutum*, the zooplanktonic larvae of the brine shrimp *Artemia franciscana,* and the marine mussel *Mytilus galloprovincialis* in order to test the hazard from phytoplankton up to a higher filter feeding trophic level. A battery of biological responses was evaluated, from growth inhibition and mortality to cytotoxicity (lysosomal membrane stability) in circulating immune cells (hemocytes) and efflux pump activity in gills. Both nanArgen™ and AgNPcitLcys were tested across a wide range of concentrations (0.001–100 mg/L) to both look for the effects of concentrations predicted to be reached in natural environmental scenarios (PEC) and find an effect threshold for nanArgen™ and AgNPcitLcys.

## 2. Materials and Method

### 2.1. Nanosilver Characterization

Nanosilver particles with citrate and L-cysteine capping (AgNPcitLcys) were synthesized by the Department of Sciences, Roma Tre University of Rome, according to Prosposito et al. [10]. NanArgen™ is a commercial brand name for a colloidal suspension of 1% *w*/*v* of PVP-capped AgNPs in MilliQ water, which was kindly supplied by Nanotek S.A. Following the procedure already described in Ale et al. [40], the nanArgen™ stock solution (10 g/L) was sonicated (30 min, 600 W, 4 kHz, 18 °C) in a sonic bath (CP316, C.E.I.A. SpA, AR, Italy) before ecotoxicity testing. The AgNPcitLcys suspension (0.6 g/L), instead, was resuspended through vortexing and sonicated at 10” in a sonic bath (CP316, C.E.I.A. SpA, AR, Italy)). The hydrodynamic diameter (<2R_H_>, nm), polydispersity index (PDI), and ζ-potential (mV) of 50 mg/L of AgNPcitLcys and nanArgen™ were measured at 25 °C in MilliQ water, F/2 (microalgal growth medium), and natural sea water (NSW) using dynamic light scattering (DLS, Zetasizer Nano ZS90, combined with Zetasizer Nano Series software, version 7.02, Particular Sciences, Malvern Panalytical, Worcestershire, UK).

### 2.2. Nanosilver Dissolution in Exposure Media

The release of Ag from AgNPcitLcys and nanArgen™ was evaluated after 1 h of incubation for both F/2 and NSW medium as well as after 72 h of incubation in F/2 and 48 h of incubation in NSW. Selected time points were chosen to mimic the exposure period: 72 h for *P. tricornutum* and 48 h for *A. franciscana* and *M. galloprovincialis*. The solutions were mixed through manual shaking once a day and kept at room temperature. At the end of the incubation time, each solution was filtered by centrifuging it for 40 min at 5000× *g* with a 3 kDa filter cut-off (Amicon Ultra-15 mL, MilliporeSigma, Burlington, MA, USA). The filtered solution was then acidified with HNO_3_ (10%).

The Ag concentrations in exposure media were determined through inductively coupled plasma–mass spectrometry (ICP-MS) using the Perkin Elmer NexION 350 spectrometer (Waltham, MA, USA). The analytical accuracy was assessed by measuring Ag concentration in the Trace Elements in Water standard reference material SRM1643e of the National Institute of Standards and Technology (NIST, Gaithersburg, MD, USA). The percentage of relative standard deviation (% RSD) of five replicate analyses of each sample was used to determine the analytical precision.

### 2.3. Microalgal Growth Inhibition Test

*P. tricornum* was used as a model marine microalgae [45], and the assay was conducted using F/2 medium prepared by adding salts to filtered NSW (0.45 μm mesh diameter). Microalgae were maintained in exponential growth conditions at 18 ± 1 °C with a 16/8 h light–dark photoperiod.

Toxicity tests were conducted using modified growth media based on F/2 medium, with adjustments made to minimize the presence of EDTA in accordance with guidelines for heavy metal toxicity assessment [46,47]. The final concentration of EDTA in the test media was 0.8 mg/L in F/2, a concentration previously demonstrated to support optimal growth of *P. tricornutum*. Microalgae from a stock culture were inoculated in the modified F/2 medium 72 h prior to testing and maintained at 21 °C under 4500 lux of continuous illumination. Single-use polystyrene multiwells with 2 mL capacity were used for the ecotoxicological assay with an initial microalgal concentration of 1 × 10^4^ cells/mL. The exposure concentrations of AgNPcitLcys, nanArgen™, and AgNO_3_ were 0, 1, 5, 10, 100, 500, and 1000 µg/L. Tests were performed in triplicate. After 72 h, the algae were fixed with a 1:1 Lugol/ethanol solution, and the cell density was determined through counting with an improved Neubauer chamber on the Olympus BX51 light microscope (Tokyo, Japan) at 40x magnification. The growth rate (μ) and the inhibition of the growth rate (Iμi) compared to the control were determined following protocol guidelines.

### 2.4. A. franciscana Immobilization Test

*A. franciscana* cysts were purchased from ECOTOX LDS (Cornaredo, MI, Italy) and kept in the dark at 4 °C until use. Then, 24 h before the test, the cysts were hydrated in a Petri dish with filtered NSW (0.22 µm) and exposed to a light source (5000 lux) until hatching. The test was performed using polystyrene multiwell with 2 mL well capacity. Each well was filled with 10 ± 2 specimens of *A. franciscana nauplii* (within 24 h from hatching, stage instar I) exposed to 0, 0.1, 1, 10, and 100 mg/L of either AgNPcitLcys, nanArgen™, or AgNO_3_. Three replicates for each concentration were set, and the multiwells were incubated at 25 °C for 48 h in the dark.

After 48 h, the surviving *nauplii* were counted under a stereomicroscope; larvae that were entirely immobile for 10 s were counted as dead, and mortality percentages were calculated. The test was replicated three times and considered acceptable if the controls had an average mortality of maximum 20%. The surviving specimens underwent rinsing with deionized water, followed by fixation in ethanol. Images were captured using the optical microscope Olympus BX51 (Tokyo, Japan) equipped with the digital camera Olympus DP-50 in conjunction with Olympus DP-SOFT software (version 3.1).

### 2.5. M. galloprovincialis In Vivo Exposure

Adult mussels of *M. galloprovincialis* (7.55 ± 0.52 cm) were purchased from an aquaculture farm (Arborea, OR, Italy) and starved for 24 h in NSW (collected from Marina di Grosseto Bay, Tuscany, NW Mediterranean Sea). Salinity and pH were measured before the tests (40.00‰, pH 8.23 ± 0.1). After 24 h of acclimatization, 3 mussels for each group were placed in a 700 mL plastic tank and exposed for 96 h to 100 µg/L of either AgNPcitLcys, nanArgen™, or AgNO_3_. A control group (only NSW) for each condition was also settled. The experiment was run in triplicate, with a total of 9 individuals for each experimental group. Exposure media were renewed after 48 h, and the organisms were not fed during exposure, according to [40]. Aeration, temperature, and photoperiod were kept constant during the exposure period (18 ± 1 °C and 18 h/6 h light/dark).

At the end of exposure time (96 h), the animals were collected and analyzed for neutral red retention time (NRRT) assay and P-glycoprotein efflux activity. The NRRT assay was conducted according to Lowe et al. [48] on mussel’s hemocytes. A sterile 1 mL syringe was used to extract the hemolymph from the mussel’s adductor muscle. The syringe was pre-loaded with 0.1 mL of buffer solution (0 mM 4-(2-hydroxyethyl)-1-piperazineethanesulfonic acid (HEPES), 436 mM NaCl, 53 mM MgSO_4_, 12 mM KCl, 10 mM CaCl_2_) used to avoid hemocyte clotting. Then, 200 mL of the obtained hemolymph suspension was placed on a coverslip (22 × 22 mm) for 1 h to allow for adhesion of a monolayer of hemocytes. After that, the hemolymph was replaced with 200 mL of neutral red dye solution (0.1 mg mL^−1^ NR in dimethyl sulfoxide, DMSO) and allowed to set for 15 min. The slides were observed under an Olympus BX51 optical microscope (Tokyo, Japan) at 80× magnification after rinsing them twice with buffer solution to remove the excess of NR. Two replicates for each group were run, and the percentage of destabilized hemocytes over 100 counted cells was recorded at 15 min intervals. Data are shown as the % of cells with destabilized lysosomal membranes over 100 cells.

The P-glycoprotein (P-gp) efflux activity was evaluated in mussels’ gills according to Neyfakh et al. [49] and modified by Luckenbach et al. [50]. Ten gill biopsies for each experimental group were incubated for 90 min in Petri dishes with 40 mL of NSW and RhB (1 µM) with gentle shaking. An additional control pool of 10 biopsies for each experimental group was exposed to NSW and RhB (1 µM) with the addition of a Pgp pump inhibitor (Verapamil 1 µM) as the positive control. After incubation, each biopsy was quickly transferred, after two rinses with NSW, to Eppendorf tubes containing 300 µL of lysis buffer (10 mM KCl, 1.5 mM MgCl_2_, 10 mM Tris pH 7.4). Using a tip Bandelin sonicator, the biopsies were sonicated (30” each, 70% of the power) and centrifuged at 13,000 rpm for 10 min. RhB fluorescence was measured using a Victor 3 1420 Multilabel Counter (PerkinElmer, Hopkinton, MA, USA) combined with the Wallac software (version 3.00) at λ_Ex_ = 522 nm, λ_Em_ = 586 nm using 96-well black microplates by counting 200 µL of the supernatant in each well. Data are expressed as Arbitrary Units of Fluorescence (AUF).

### 2.6. Statistical Analysis

Statistical analyses were carried out with GraphPad version 8 (GraphPad Software, La Jolla, CA, USA). EC_50_ values for the growth inhibition test and the immobilization test were calculated using non-linear regression analysis, while statistical significance between mean values was calculated with a non-parametric test (Kruskal–Wallis test) with Dunn’s correction for multiple comparisons. Data are expressed as the mean ± standard deviation (SD).

The statistical analysis of the biomarker data was conducted utilizing R and R-Studio software (version 2024.12.1+563). The Kruskal–Wallis ANOVA test was employed to analyze the efflux pump response, whereas the *t*-test was used to assess lysosomal membrane stability (LMS). The resulting data are expressed as mean ± standard deviation (SD).

## 3. Results and Discussion

### 3.1. Behavior of Nanosilver Formulations in Exposure Media

Physicochemical characterization of AgNPcitLcys and nanArgen™ in MilliQ water, F/2, and NSW is reported in Table 1. DLS analysis reveals a negative ζ-potential in MilliQ water for both AgNPcitLcys and nanArgen™, which increases upon suspension in F/2 and NSW, remaining negative. The hydrodynamic diameter (<2R_H_>), which was slightly higher for nanArgen™, is around 200 nm for both NPs, but PDI values suggest the presence of different size populations, highlighting a moderate aggregation of the particles already in MilliQ water. Both F/2 and NSW cause a clear aggregation of AgNPcitLcys and nanArgen™; however, especially in NSW, the size population seems to be narrowed around the measured <2R_H_> value, as suggested by the lower PDI values (0.27 and 0.29 respectively).

<2R_H_> and ζ-potential values of nanArgen™ and AgNPcitLcys in MilliQ water and NSW confirm our previous findings [30,40] as a confirmation of the good preservation of both batches over time.

The observed formation of large aggregates in NSW and F/2 is probably caused by their high ionic strength, which generates the compression of the electric double layer of the NP surface and the reduction of repulsive electrostatic forces between the NPs [51,52].

**Figure 1:**
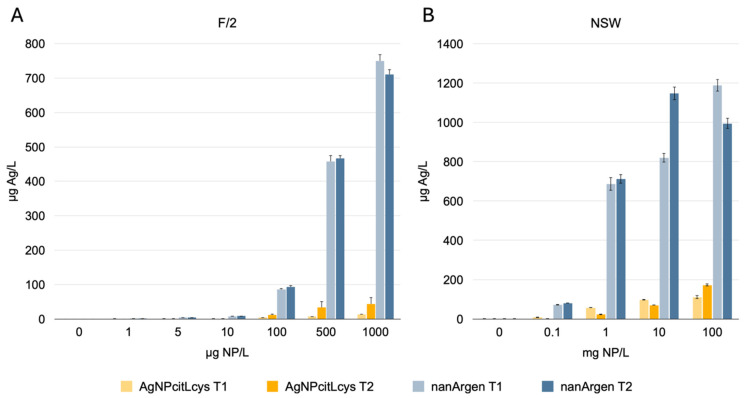
Ag concentration (µg/L) in (**A**) F/2 and (**B**) NSW upon incubation of AgNPcitLcys and nanArgen™ at, respectively, 0, 1, 5, 10, 100, 500, and 1000 µg/L and 0, 0.1, 1, 10, and 100 mg/L of AgNPs. Results for 1 h of incubation (T1), 72 h of incubation for F/2, and 48 h of incubation for NSW (T2). Data shown as mean ± standard deviation.

Dissolution data reveal a great difference between AgNPcitLcys and nanArgen™, with the eco-designed NPs being less prone to releasing Ag than the NP in the commercial formulation, both in F/2 and NSW (Figure 1). The maximum release of Ag (172.92 µg/L) by AgNPcitLcys is reached only at the very high concentration of 100 mg/L after 1 h of incubation in NSW, while nanArgen™ showed the highest dissolution values of 1200 µg/L and 700–800 µg/L in, respectively, NSW and F/2.

Such low dissolution for AgNPcitLcys in MilliQ water and NSW has already been observed and reported in previous work [10,30,31]. Such behavior was attributed to the L-cysteine groups in the coating acting either as Ag ions chelators or by effectively protecting the NP silver surface from oxidizing agents.

Conversely, nanosilver in the commercial formulation nanArgen™ has strong dissolution behavior similar to what is reported in the literature for PVP-coated AgNPs [33,53]. However, for both NPs, there is not much difference from the amount of Ag released after 1 h and 48–72 h of incubation, showing that the dissolution process is fast and more influenced by the coating agent than the incubation period.

### 3.2. Ecotoxicity Tests

A summary of the ecotoxicological results (Tables S1 and S2) is available in the Supporting Information.

#### 3.2.1. Microalgal 72 h Growth Inhibition Test with *P. tricornutum*

**Figure 2:**
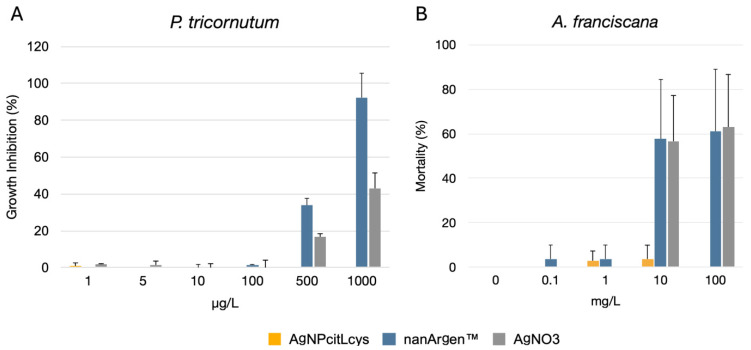
(**A**) 72 h growth inhibition test of AgNPcitLcys (yellow), nanArgen™ (blue), and AgNO_3_ (gray) at 0, 1, 5, 10, 100, and 1000 µg/L with *P. tricornutum*; (**B**) 48 h mortality test of AgNPcitLcys (yellow), nanArgen™, (blue) and AgNO_3_ (gray) at 0, 0.1, 1, 10, and 100 mg/L with *A. franciscana*. Data shown as mean ± standard deviation.

As shown in Figure 2A, AgNPcitLcys caused no effect on *P. tricornutum* growth up to the highest tested concentration (1000 µg/L). Conversely, nanArgen™ caused a 35% inhibition of growth at 500 µg/L and almost 100% inhibition at 1000 µg/L. Such findings agree with our previous studies in which AgNPcitLcys [10,30,31] was far less toxic to microalgae compared to other AgNPs with different coatings. The protective role of the eco-designed coating made of citrate and L-cysteine might explain such results by reducing the dissolution of the nanosilver and the release of Ag. This can be mainly attributed to the reduced sulfur groups possessed by L-cysteine molecules, shielding the particle’s surface from oxidation and preventing ion release [54,55]. At the same time, a significant role in reducing nanosilver toxicity might also be played by the diatom siliceous cell wall. In fact, AgNPcitLcys toxicity was previously hypothesized to be mainly driven by membrane adhesion and/or cell internalization, instead of being caused by ion release [31]. In this case, the siliceous wall might protect the cells by preventing AgNPcitLcys adhesion to the cell membrane, limiting possible damages, and avoiding the occurrence of direct toxicity caused by AgNP contact. The protective role of the siliceous wall of *P. tricornutum* against AgNP toxicity has already been hypothesized in a study by Schiavo et al. [56] showing lower toxic effects for the diatom compared to microalgae without a cell wall.

Conversely, high toxicity is observed for nanArgen™, which is characterized by significant dissolution upon suspension in water media, leading us to identify dissolution as the main mechanism of toxicity of nanArgen™. This agrees with what was reported by Tsiola et al. [57] when describing high toxicity for PVP-coated AgNPs, which the authors ascribe to the high rate of dissolution. Similar results are presented by Angel et al. [58], who state that for PVP-capped AgNPs, released silver is a better predictor of toxicity to microalgae compared to AgNP’s nominal concentration.

However, the particulate nature of the nanArgen™ formulation seems to bring an additive toxicity to that caused by dissolved Ag ions, as its toxicity is greater compared to that caused by AgNO_3_ (Figure 2A), even though the difference is not statistically significant. The same was observed by Andrade et al. [17], who investigated the toxicity of nanArgen™ to the cladoceran *Ceriodaphnia reticulata* and how the presence of the microalgae *Tetradesmus obliquus* modified such toxicity. The results showed that nanArgen™ toxicity was more pronounced compared to that of AgNO_3_, but it was mitigated by the presence of *T. obliquus*, even though the presence of the algae increased ion release. This further confirms our hypothesis of an additive effect beyond ion release playing a role in nanArgen™ toxicity.

#### 3.2.2. *A. franciscana* Immobilization Test

**Figure 3:**
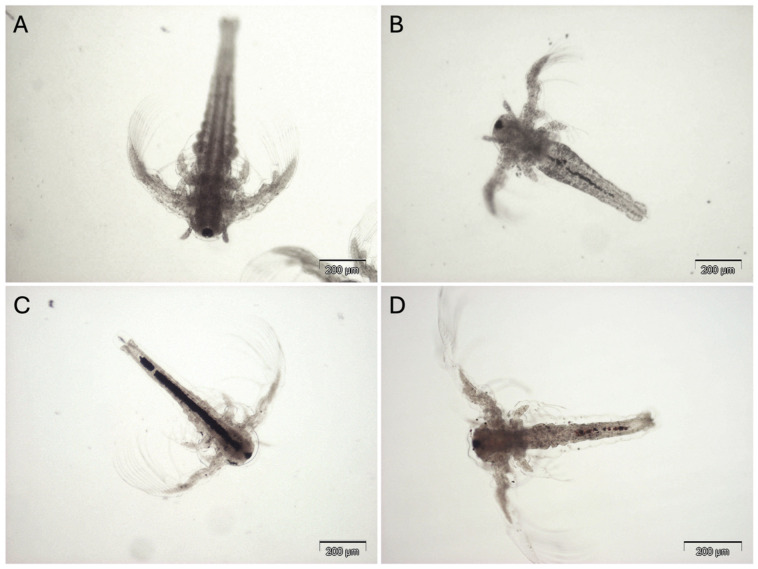
*A. franciscana* specimens after 48 h of exposure to (**A**) CTRL, (**B**) 100 mg/L of AgNO_3_, (**C**) 100 mg/L of AgNPcitLcys, and (**D**) 100 mg/L of nanArgen™.

Similarly to what we observed for microalgae, acute exposure of *A. franciscana* confirms low toxicity of the eco-designed AgNPcitLcys and high toxicity of nanArgen™ at high concentrations (10 and 100 mg/L with 59.9 ± 26% and 61 ± 28% mortality, respectively) (Figure 2B). These findings agree with the literature reporting concentration-dependent mortality of *Artemia salina* exposed to different concentrations of PVP-capped AgNPs [59]. Again, the obtained results can be attributed to the low rate of dissolution of AgNPcitLcys and, on the other hand, the high release rate of ions from nanArgen™. This might also be confirmed through morphological observation, as *A. franciscana* exposed to nanArgen™ shows slight malformations of the external cuticle (Figure 3B), which is similar to those exhibited by AgNO_3_-exposed specimens (Figure 3D). A recent study by de Paiva Pinheiro et al. [60] showed how Ag ions can bind to different functional groups of the chitin molecules, the main structural component of *Artemia*’s cuticle, inducing structural changes in the chitin layer. This might also facilitate AgNP and Ag ions’ diffusion inside of the cuticle and accumulation inside of the cells, with further detrimental effects to the organisms.

Interestingly, ingestion does not seem to play a determinant role in the observed toxicity, as it appears to be much more pronounced for AgNPcitLcys (Figure 3C) compared to nanArgen™ (Figure 3B). Instead, ingestion might have exacerbated nanArgen™ toxicity by enhancing dissolution inside of the animal’s gut, as mortality is higher than expected compared to measured dissolved Ag. This difference is probably driven by the different cappings, which might react differently to the gut’s environment, once again confirming the protective role of the -citLcys coating in reducing AgNPcitLcys toxicity. A similar scenario was reported by Demarchi et al. [61], who confronted the effects of various AgNPs with different coating agents and reported ingestion coupled with different levels of mortality and oxidative stress, highlighting the different levels of protection of some cappings compared to others.

#### 3.2.3. *M. galloprovincialis* In Vivo Exposure

At the end of the exposure period (96 h), 11% mortality was observed in control organisms, while an increase in mortality was observed for AgNO_3_ (22%), AgNPcitLcys at 100 µg/L (33%), and nanArgen™ (44%). Here, a mix of effects due to the nano size and Ag ions is hypothesized, as the lethality is double for the commercial products compared to AgNO_3_, while an intermediate value is obtained for the eco-designed AgNPcitLcys. Moreover, the PVP coating from nanArgen^TM^ could have generated an additional toxic effect, as its higher toxicity in terms of mortality for early stages of *Oryzias latipes* fish when compared with other kinds of coatings has been reported [32].

**Figure 4:**
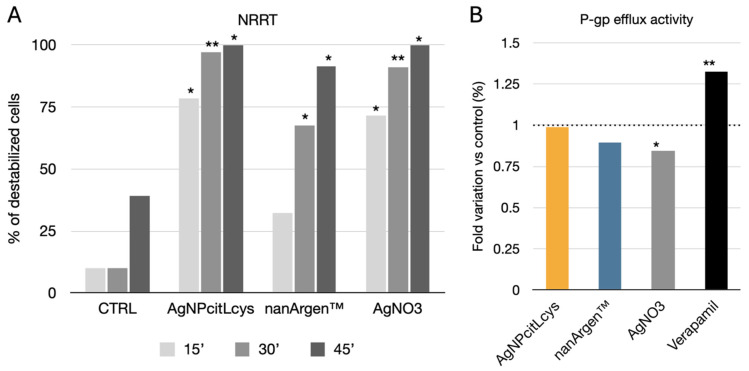
(**A**) NRRT expressed as % of destabilized cells of mussels exposed to 100 µg/L of AgNPcitLcys, nanArgen™, and AgNO_3_. Values are expressed as means ± SD out of 100 hemocytes scored. Asterisks above the bars show significant differences with control groups at each time point (* *p* < 0.05; ** *p* < 0.01). (**B**) P-gp efflux activity in gills of mussels exposed to 100 µg/L of AgNPcitLcys, nanArgen™, and AgNO_3_. Verapamil (VER, 1 µM) is shown as the positive control. Data shown as fold variation in RhB content compared to controls. Asterisks on the above the bars show significant differences with the respective control groups (* *p* < 0.05; ** *p* < 0.01).

As far as lysosomal membrane stability (LMS) measured in mussels’ hemocytes, all silver forms increased the percentage of lysosomal destabilization (Figure 4A). A time-dependent trend was observed because at 15 min no destabilization was observed in lysosomes of mussel exposed to nanArgen™, while both AgNPcitLcys and AgNO_3_ caused a significant increase in destabilization compared to the controls (CTRL) (*p* < 0.05) and to a similar extent. After 30 min, nanArgen™ caused significant destabilization compared to the controls (*p* < 0.05), but it was lower than that observed for AgNPcitLcys and AgNO_3_ (*p* < 0.05). After 45 min, both the nanosilver formulation and AgNO_3_ caused significant destabilization compared to the controls.

Overall, AgNPs are widely reported to disrupt LMS by promoting superoxide anion generation (and, consequently, generating lipid peroxidation), interacting with membrane thiol groups (-SH), and, thus, interfering with protons in the intermembrane space, thereby potentially affecting the electron flow [62]. Our findings agree with previous results reporting a time-dependent increase in LM destabilization upon mussel exposure to nanArgen™ [40]. Similarly, Katsumiti et al. [63] reported the disruption of LMS in mussels’ hemocytes regardless of the silver form (AgNPs, bulk silver, and ionic silver).

Based on these data, the effect of either AgNP or Ag ions would barely be distinguishable in affecting the LM of circulating hemocytes, probably because once inside of the cells they would both end up in lysosomes, causing membrane destabilization. In this regard, the “Trojan Horse” effect has been proposed as one of the main mechanisms of cytotoxicity as the particles are able to cross the cell membranes and, once inside of the cells, Ag ions are released [64]. Previous findings showed the affinity of AgNPcitLcys for biomolecules and their ability to develop a protein corona when dispersed in protein-rich media thanks to the citrate and L-cysteine capping [31]. This affinity might enhance AgNPcitLcys interaction with *M. galloprovincialis* hemocytes, explaining the greater disruption of LMS observed during AgNPcitLcys exposure. It would be interesting to track AgNP’s fate once it is internalized by cells to unravel their stability and/or dissolution into the cytosol and link it to the observed toxicity in organelles.

Concerning the efflux pump response, no effects were observed (Figure 4B) in mussels exposed to AgNPcitLcys and nanArgen™, while a slight increase in RhB content (decrease in P-gp efflux) was observed in mussels exposed to AgNO_3_ vs. the control (*p* < 0.05). Verapamil caused a significant increase in RhB content vs. all experimental groups, confirming its role as an ABC pump inhibitor [65]. The absence of effects on the P-gp pump for both nanArgen™ and AgNPcitLcys seems to be consistent with our previous findings, in which 10 µg/L of nanArgen™ did not cause increased Ca-AM efflux activity in *M. galloprovincialis* [40]. Accordingly, it has been explained that AgNPs could penetrate the membranes via pore canals, with uptake kinetics characteristic of diffusion rather than active transport [66]. However, although gills are the first line of defense of filter feeders, they do not seem to be targets for Ag accumulation [21].

The observed results suggest that the accumulation of Ag occurred mainly in the lysosomal compartment rather than in the gills. Mussel gills are rich in mucocytes that secrete a solution mainly composed of acidic mucopolysaccharides [67] acting as a food trap, including particles [68,69]. Those acidic mucopolysaccharides might be stimulating the extracellular dissolution of nanosilver, increasing Ag uptake by the mussels and resulting in enhanced LM destabilization. Another hypothesis might be formulated; considering that ion release from AgNPcitLcys is virtually absent in NSW, the different toxicity between AgNPcitLcys and nanArgen™ might be linked to the different particles’ nominal sizes, 12.5 ± 1 nm and 30 ± 10 nm, respectively, as shown in our previous work [31,40]. The smaller size of the eco-designed AgNPcitLcys, together with the aforementioned affinity for biomolecules, might have enhanced cell internalization and facilitated particle transport in the hemolymph through the gills, as opposed to nanArgen™. Such a hypothesis is supported by our previous findings on AgNPcitLcys’s toxic mode of action, which appears to be more nano-size related compared to other AgNPs [31], and it is corroborated by the findings reported by Bouallegui et al. [70], who found that 50 nm sized AgNP caused greater destabilization of LMs compared to 100 nm sized ones. On the contrary, from the comparison of the amount of Ag released by nanArgen™ and the effect of AgNO_3_ exposure, nanArgen™ toxicity to *M. galloprovincialis* seems to be solely driven by dissolution and ion release.

## 4. Conclusions

The present study investigates the behavior and marine ecotoxicity of nanosilver in the commercial product nanArgen™ and the eco-designed bifunctionalized formulation (AgNPcitLcys) on microalgae, microcrustaceans, and mussels. Both AgNPs were observed to form aggregates in NSW, but dissolution and Ag release were more marked for nanArgen™ compared to AgNPcitLcys (at least one order of magnitude lower). No toxicity was observed for microalgae and microcrustaceans for AgNPcitLcys, while nanArgen™ was toxic at high concentrations to both of them (≥500 µg/L for *P. tricornutum* and ≥10 mg/L for *A. franciscana*). Ag release played a key role in nanArgen™ toxicity, even though, from the comparison with AgNO_3_, the occurrence of an intrinsic additive nano-form toxicity besides that of ion release could be hypothesized.

Lethal effects were observed in the marine mussel *M. galloprovincialis* upon exposure to both nanArgen™ and AgNPcitLcys, which were higher for the latter and also associated with significant time-dependent destabilization of lysosomal membranes. This was attributed to the smaller nominal size of AgNPcitLcys, which might have enhanced internalization inside of mussel cells and toxicity. Therefore, nanArgen™ toxicity to *M. galloprovincialis* seemed to be solely driven by dissolution and ion release and barely associated with the nano-form.

Overall, the results showed the potentially high environmental impact of nanosilver-enabled consumer products, but they also showed how organisms with different life strategies can differently suffer from exposure to the same AgNP. Moreover, this study confirmed how surface capping is the main element driving AgNP behavior and toxicity, highlighting the importance of acting on particles’ eco-design in order to control their environmental impact.

## Figures and Tables

**Table 1:** Hydrodynamic diameter (<2R_H_>) by intensity, polydispersity index (PDI), and ζ-potential values of 50 mg/L of AgNPcitLcys and 50 mg/L of nanArgen™ measured in MilliQ water, F/2, and NSW at 25 °C.

		<2R_H_> (nm) Intensity	PDI	ζ-Potential (mV)
AgNPcitLcys	MilliQ	179 ± 30	0.5	−48 ± 4
	F/2	560 ± 16	0.7	−8 ± 2
	NSW	773 ± 61	0.29	−4 ± 1
nanArgen™	MilliQ	226 ± 9	0.4	−14 ± 1
	F/2	931 ± 34	0.28	−6 ± 3
	NSW	931 ± 20	0.27	−7 ± 1

## Data Availability

The raw data supporting the conclusions of this article will be made available by the authors upon request.

## Supplementary Materials

The following supporting information can be downloaded at: https://www.mdpi.com/article/10.3390/toxics13050338/s1, Table S1: Summary of toxicity data obtained from the exposure of *P. tricornutum* and *A. franciscana* to different concentrations of AgNPcitLcys, nanArgen™ and AgNO_3_. Data are expressed as percentage of growth inhibition compared to control for *P. tricornutum* and percentage of mortality for *A. franciscana.* Data are shown as mean ± standard deviation.; Table S2: Summary of toxicity data obtained from the exposure of *M. galloprovincialis* to 100 µg/L of AgNPcitLcys, nanArgen™ and AgNO_3_. Data are expressed as percentage of mortality, percentage of destabilized cells after 15, 30 and 45 minutes for NRRT assay and fold variation *versus* control for the Pgp activity.
