# Supplementary material for: Nanosilver Environmental Safety in Marine Organisms: Ecotoxicological Assessment of a Commercial Nano-Enabled Product vs an Eco-Design Formulation
Source: Toxics. 2025 Apr 25;13(5):338. doi: 10.3390/toxics13050338 (PMC12115498; doi:10.3390/toxics13050338)
Supplement: Supplementary file 1 [file toxics-13-00338-s001.zip › toxics-3566093-supplementary.pdf]

Supporting information – Nanosilver environmental safety in marine organisms: ecotoxicological assessment of a commercial nano-enabled product *vs* an eco-design formulation

Table S1. Summary of toxicity data obtained from the exposure of *P. tricornutum* and *A. franciscana* to different concentrations of AgNPcitLcys, nanArgen™ and $AgNO_3$. Data are expressed as percentage of growth inhibition compared to control for *P. tricornutum* and percentage of mortality for *A. franciscana*. Data are shown as mean ± standard deviation.

Conc. (mg/L)	*P. tricornutum*			*A. franciscana*		
	Growth inhibition compared to control (%)			Mortality (%)		
	AgNPcitLcys	nanArgen™	$AgNO^3$	AgNPcitLcys	nanArgen™	$AgNO^3$
CTRL	---	---	---	0	0	0
0.001	0	0	1.7 ± 0.3	---	---	---
0.005	0	0	1.3 ± 2.6	---	---	---
0.01	0	0	0	---	---	---
0.1	0	1.4 ± 0.6	0	0	3.7 ± 6	0
0.5	0	33.8 ± 3	16.8 ± 2	---	---	---
1	0	92 ± 13	43.1 ± 8	2.7 ± 4	3.7 ± 6	0
10	---	---	0	3.7 ± 6	57.9 ± 26	56 ± 20
100	---	---	---	0	61± 28	63 ± 24

Table S2. Summary of toxicity data obtained from the exposure of *M. galloprovincialis* to 100 µg/L of AgNPcitLcys, nanArgen™ and $AgNO_3$. Data are expressed as percentage of mortality, percentage of destabilized cells after 15, 30 and 45 minutes for NRRT assay and fold variation *versus* control for the Pgp activity.

M. galloprovincialis	Mortality (%)	NRRT Destabilized cells (%)			Pgp activity Fold variation *vs* control
		15’	30’	45’	
CTRL	11%	10%	10%	39%	---
AgNPcitLcys (100 µg/L)	33%	78.5%	97%	100%	0.988
nanArgen™ (100 µg/L)	44%	32.5%	67.5%	91.5%	0.894
$AgNO_3$ (100 µg/L)	22%	71.5%	91%	100%	0.843
